# Heart Rate Variability in Women with Systemic Lupus Erythematosus: Association with Health-Related Parameters and Effects of Aerobic Exercise

**DOI:** 10.3390/ijerph17249501

**Published:** 2020-12-18

**Authors:** Elena Martínez-Rosales, Sergio Sola-Rodríguez, José Antonio Vargas-Hitos, Blanca Gavilán-Carrera, Antonio Rosales-Castillo, Alba Hernández-Martínez, Enrique G. Artero, José Mario Sabio, Alberto Soriano-Maldonado

**Affiliations:** 1 Department of Education, Faculty of Education Sciences, University of Almería, 04120 Almería, Spain; sergiosola95@gmail.com (S.S.-R.); albaherzm@ual.es (A.H.-M.); artero@ual.es (E.G.A.); asoriano@ual.es (A.S.-M.); 2 SPORT Research Group (CTS-1024), CERNEP Research Center, University of Almería, 04120 Almería, Spain; 3 Systemic Autoimmune Diseases Unit, Department of Internal Medicine, Virgen de las Nieves University Hospital, 18014 Granada, Spain; joseantoniovh@hotmail.com (J.A.V.-H.); anrocas90@hotmail.com (A.R.-C.); jomasabio@gmail.com (J.M.S.); 4 Physical Activity for Health Promotion Research Group (PAHELP), Sport and Health University Research Institute (iMUDS), Department of Physical Education and Sports Faculty of Sport Sciences, University of Granada, 18071 Granada, Spain; bgavilan@ugr.es

**Keywords:** autonomic nervous system, exercise, inflammation, fatigue, rheumatic disease

## Abstract

Abnormal heart rate variability (HRV) has been observed in patients with systemic lupus erythematosus (SLE). In a combined cross-sectional and interventional study approach, we investigated the association of HRV with inflammation and oxidative stress markers, patient-reported outcomes, and the effect of 12 weeks of aerobic exercise in HRV. Fifty-five women with SLE (mean age 43.5 ± 14.0 years) were assigned to either aerobic exercise (*n* = 26) or usual care (*n* = 29) in a non-randomized trial. HRV was assessed using a heart rate monitor during 10 min, inflammatory and oxidative stress markers were obtained, psychological stress (Perceived Stress Scale), sleep quality (Pittsburg Sleep Quality Index), fatigue (Multidimensional Fatigue Inventory), depressive symptoms (Beck Depression Inventory), and quality of life (36-item Short-Form Health Survey) were also assessed. Low frequency to high frequency power (LFHF) ratio was associated with physical fatigue (*p* = 0.019). Sample entropy was inversely associated with high-sensitivity C-reactive protein (*p* = 0.014) and myeloperoxidase (*p* = 0.007). There were no significant between-group differences in the changes in HRV derived parameters after the exercise intervention. High-sensitivity C-reactive protein and myeloperoxidase were negatively related to sample entropy and physical fatigue was positively related to LFHF ratio. However, an exercise intervention of 12 weeks of aerobic training did not produce any changes in HRV derived parameters in women with SLE in comparison to a control group.

## 1. Introduction

Systemic lupus erythematosus (SLE) is a systemic autoimmune disease with multifactorial etiology that predominantly affects women [1]. In recent years, the diagnosis and treatment of SLE has significantly improved [2], and deaths due to lupus manifestation have decreased [3]. However, cardiovascular disease (CVD) mortality remains one of the leading causes of death in SLE patients [4,5].

The importance of the autonomic nervous system (ANS) on cardiovascular health and prognosis has already been reported [6,7]. In fact, the ANS plays a key role in regulating immune responses to inflammatory stimuli [8]. Heart rate variability (HRV) is a noninvasive and sensitive measure of ANS function [9] and is defined as the physiological variation in the duration of intervals between sinus beats [10]. Autonomic dysfunction is common in autoimmune rheumatic diseases [11], and specifically, increased sympathetic and decreased parasympathetic activity as reported by several studies in patients with SLE [12,13,14]. In this sense, patients with SLE have shown abnormal HRV, a surrogate marker of cardiac ANS dysfunction [15], which may predispose to the onset of fatal arrhythmias in these patients [16]. Considering that HRV is inversely associated with inflammatory markers in healthy individuals and in patients with CVD [17], it is of clinical interest to: (i) understand the extent to which HRV might be associated to inflammatory markers and patient-reported outcomes (PROs) and (ii) whether HRV can be enhanced through interventions in women with SLE.

Exercise is a potential intervention that significantly increases cardiorespiratory fitness [18,19], improves cardiovascular function and PROs (i.e., fatigue, depression, etc.) [20] in patients with SLE. Although exercise has shown to decrease cardiovascular morbidity and mortality in the general population [21,22], its benefits in SLE population are understudied to the extent that exercise hardly appear in the EULAR guidelines for the management of this chronic disease [23]. Benatti and Pedersen [24] suggested that one of the mechanisms by which exercise might benefit the cardiovascular system in patients with rheumatic diseases is through direct or indirect anti-inflammatory effects. Based on the effects of exercise in the general population [25] and other chronic conditions [26,27], it might be hypothesized that exercise (and particularly aerobic exercise) could also increase HRV and thus regulate the ANS in women with SLE. Although there have been some studies evaluating HRV after an exercise stress test in this population [28,29], to the best of our knowledge, no prior research has evaluated the effects of an aerobic exercise program on HRV in women with SLE.

Therefore, the aims of this study are (1) to cross-sectionally explore the associations of HRV with inflammatory markers and PROs; and (2) to analyze the effect of a 12-week aerobic program in women with SLE on HRV derived parameters.

## 2. Materials and Methods

### 2.1. Study Design and Participants

This study included data of 58 women with SLE from a non-randomized controlled trial investigating the effects of a 12-week aerobic exercise program on arterial stiffness, inflammation, and cardiorespiratory fitness [19]. Participants were recruited from the Systemic Autoimmune Diseases Unit of the “Virgen de las Nieves” and “San Cecilio” University Hospitals (Granada, Spain). A comprehensive description of the inclusion and exclusion criteria can be found elsewhere [19]. The study was approved by the Research Ethics Committee of Granada (ref. No.: 10/2016) and registered at clinicaltrials.gov [NCT03107442] with HRV among the pre-established secondary outcomes. All participants signed written informed consent. The baseline data were used for the cross-sectional analyses of the present study.

### 2.2. Intervention

#### 2.2.1. Exercise Group

The exercise program has been comprehensively described elsewhere [19] following the Consensus on Exercise Reporting Template (CERT) [30]. Participants assigned to the exercise group performed two 75-min sessions per week of moderate to vigorous intensity aerobic exercise on a treadmill (BH, Serie i.RC12 Dual, Vitoria-Gasteiz, Spain) for 12 weeks. All sessions began with a warm-up on the treadmill at about 35–40% of the heart rate reserve (HRR) plus 3–4 min of active stretching, while ending with a cool down of static stretching and relaxation. Exercise was prescribed with training intensity progressively increasing in a range from 40% to 75% of each individual’s HRR. In all sessions, heart rate was monitored with a Polar V800 (Polar Inc., Kempele, Finland).

Only continuous exercise was performed during the first half of the program. Continuous sessions comprised several bouts of exertion at constant intensity, followed by a couple of minutes of recovery. At 8 weeks, continuous and interval sessions were alternated, and at 12 weeks, the patients performed only interval training sessions, with periods of lower and higher intensity efforts followed by some minutes of rest for hydration. The progression in volume and/or intensity was undertaken by increasing the treadmill speed or inclination according to the perceived exertion of each patient. Lastly, the exercise intensity progressions had to be slightly modified since several patients perceived a 5% HRR intensity increase as very heavy and difficult-to-follow. Therefore, exercise intensity increased by 2.5% instead of 5% in some weeks.

#### 2.2.2. Control Group

SLE patients assigned to the control (usual care) group received information about a healthy lifestyle, including physical activity guidelines and basic nutritional information.

### 2.3. Heart Rate Variability

Participants were requested not to drink caffeinated or alcoholic drinks, to fast for at least 3 h, and not to participate in physical activity 24 h before the assessment. R-R intervals were recorded with a Polar V800 (Polar Inc., Kempele, Finland), a validated instrument [31], placed at the sternum level. Participants were place in supine position in a quiet room (temperature 22–24 °C) between 4 p.m. and 7 p.m., and were instructed to breath normally, stay relaxed and not to speak or fidget during the assessment. HRV was recorded for 10 min, after a period of 5 min, at a sampling frequency of 1000 Hz. HRV raw data was analyzed with Kubios (HRV analysis, Finland). After visual inspection for any premature contractions or ectopic beats in the recording, a 5-min period was manually selected by the evaluator. Kubios filters were applied accordingly based on inter-individual variability and if the sample presented more than 5% of interpolated R-R intervals it was discarded as per manufacturer’s recommendation [32].

The following HRV derived parameters were analyzed: the standard deviation of the average normal-to-normal (NN) interval (SDNN), the square root of the mean squared differences of successive NN intervals (RMSSD), and percentage of consecutive R-R intervals that differ by more than 50 ms (pNN50), low frequency power (LF: 0.04–0.15 Hz), high frequency power (HF: 0.15–0.4 Hz) and LF to HF power ratio (LFHF) indices (which were computed using the fast Fourier transform), Poincaré Plot were standard deviation 1 (SD1), represents short-term variability, and standard deviation 2 (SD2), the long-term variability (compared with SD1); and sample entropy (SampEn).

### 2.4. Patient-Reported Outcomes

Health-related quality of life was assessed using the short version of the Spanish version of the 36-item Short-Form Health Survey (SF-36) [33]. Depression was assessed through the Beck Depression Inventory-second edition (BDI-II) [34]. Psychological stress was measured with the Perceived Stress Scale (PSS) [35], and fatigue with the Multidimensional Fatigue Inventory (MFI) [36].

### 2.5. Inflammatory and Oxidative Stress Markers

Fasting blood samples for biochemical and immunological tests were collected and processed. High-sensitivity CRP (hsCRP), interleukin 6 (IL-6), and tumor necrosis factor α (TNF-α) were measured as markers of inflammation, whereas myeloperoxidase (MPO) was determined as a marker of oxidative stress.

### 2.6. Other Measurements

Height was measured using a height gauge, weight with a bioimpedance device (InBody R20, Korea), and body mass index (BMI) was calculated (kg/m^2^). Blood pressure was measured with Mobil-O-Graph^®^ (IEM GmbH, Stolberg, Germany) [37]. Disease activity was assessed through the Systemic Lupus Erythematosus Disease Activity Index (SELENA-SLEDAI) [38]. Physical activity was self-reported with the International Physical Activity Questionnaire [39]. All participants filled out a socio-demographic and clinical data questionnaire.

### 2.7. Classification of Responders, Non-Responders, and Adverse Responders

The inter-individual variability of the patients in the response to the intervention was analyzed by categorizing participants from each group as responders, non-responders or adverse responders using the typical error measurement (TE). The TE was calculated using the equation TE = SDdiff/√2, where SDdiff is the standard deviation of the difference scores observed between the 2 repeats of each measurement [40]. A responder was defined as an individual who demonstrated an increase (in favor of beneficial changes), an adverse responder was defined as an individual who demonstrated a decrease, and a non-responder was defined as an individual who failed to demonstrate an increase or decrease that was >2 times the TE away from 0. A change more than 2 times the TE means that this response is a true physiological adaptation beyond what might be expected to result from technical and/or biological variability [41].

### 2.8. Treatment Allocation and Blinding

Randomization was not possible as many participants lived far and were not able to attend the exercise sessions in case of being randomized to exercise. Therefore, participants from the city of Granada were included in the exercise group and participants living outside Granada were included in the control group. To minimize potential selection bias, we aimed to match the groups by age (±2 years), BMI (±1 kg/m^2^), and SLEDAI (±1 unit). The data analyzer was blinded to the patient allocation.

### 2.9. Statistical Analysis

Normality was tested using visual inspection of histograms and Q-Q plots. As HRV-derived parameters were non-normally distributed, their descriptive analysis was presented using median and interquartile range, while non-parametric test was used for the main analysis. Between-group baseline characteristics were compared with the Student t-test (when normally distributed), Kruskal–Wallis test (when non-normally distributed) for continuous variables and the Chi-square test for categorical variables. To explore the associations of HRV with inflammatory and oxidative stress markers (hsCRP, IL-6, TNF-α and MPO) and PROs (aim 1), scatter plots and Spearman’s bivariate correlations were used as preliminary analyses to understand raw associations. Subsequently, quantile regression models were built, including each of the above HRV parameters as dependent variables and each inflammatory marker as independent variables in regression models along with age, heart rate, and disease duration as relevant factors that might confound the association of interest. This same procedure was followed with PROs. Other variables included in the regression model were SLEDAI, systemic damage index (SDI), and smoking. However, neither of these variables affected the regression coefficients; therefore, they were not included. Inflammatory markers (hsCRP, IL-6 and TNF–α) and MPO were winsorized to the highest value due to the presence of outliers.

To assess the effects of the exercise intervention (aim 2), the between group differences in the change from baseline in HRV-derived parameters were assessed through quantile regression with baseline values, heart rate, and age as covariables. As we aimed at assessing efficacy, the primary analyses were defined as per-protocol, where patients from the exercise group were included if attendance to the exercise sessions was ≥75%. We additionally performed sensitivity analyses including (i) participants with attendance ≥90%; and (ii) baseline observation carried forward (BOCF). All the analyses were conducted with SPSS v.26 (IBM SPSS Statistics, Chicago, IL, USA). Statistical significance was set at *p* < 0.05.

## 3. Results

**Figure 1:**
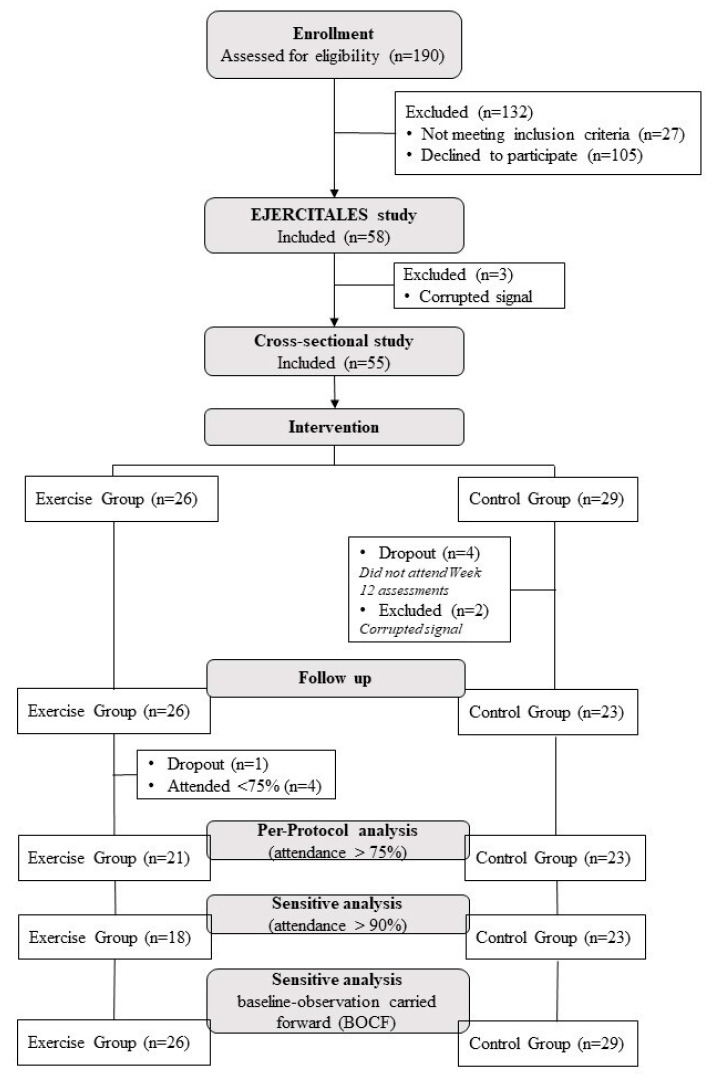
Flowchart of the study participants throughout the study.

The flowchart of the study participants throughout the trial is presented in Figure 1. A total of 58 patients completed the baseline assessment and were included in aim 1 analysis (*n* = 55).

For aim 2, participants were assigned to either the exercise group (*n* = 26) or the control group (*n* = 32). At baseline (Table 1 and Table 2), the control group showed a higher IL-6 levels (median difference 3.10 pg/mL; *p* = 0.018), lower score in the physical component summary of the SF-36 (mean difference −4.9 units; *p* = 0.034), and higher punctuation in depressive symptoms (mean difference 9.0 units; *p* = 0.011) than the exercise group.

### 3.1. Associations of HRV with Inflammatory, Oxidative Stress Markers, and PROs (Aim 1)

The raw association of the HRV parameters with inflammatory markers and PROs is presented in abbreviated form in Table 3 (see Table S1 and Figure S1 for more details). SampEn was inversely correlated with hsCRP and MPO (r = −0.35, *p* < 0.01 and r = −0.32, *p* < 0.05, respectively). LFHF ratio was positively correlated with IL-6 (r = 0.32, *p* < 0.05). There was no association of any time-domain derived parameter with inflammatory markers. Regarding PROs, LFHF ratio was positively correlated with the Physical Fatigue dimension of the MFI (r = 0.30, *p* < 0.05). There were no other significant correlations.

The quantile regression models evaluating the association between HRV parameters, inflammatory markers, and PROs are presented in Table 4 adjusted by age, heart rate and disease duration. Only significant correlations were explored. LFHF ratio was associated with the physical fatigue dimension of the MFI (unstandardized coefficient (B) = 0.89; 95% confidence interval (CI) 0.15 to 1.62; *p* = 0.019) but there was no association with IL-6 (B = 0.48; 95% CI −0.31 to 1.27; *p* > 0.05). SampEn was inversely associated with hsCRP (B = −4.82; 95% CI −8.62 to −1.03; *p* = 0.014) and MPO (B = −106.51; 95% CI −182.54 to −30.50; *p* = 0.007). We did not find associations of HRV derived parameters with SLEDAI or SDI.

### 3.2. Effects of the Exercise Intervention on HRV-Derived Parameters (Aim 2)

The HRV signals from 5 participants from the control group were excluded due to excessive interpolated beats (>5%). Full HRV data at baseline and week 12 was obtained from 44 participants (21 exercise and 23 control). The primary analyses revealed no significant between-group differences between changes in HRV derived parameters (Table 5) in all domains, and these results were consistent in sensitivity analyses in which participants from the exercise group were included only when attendance of the exercise sessions was ≥90% (Table S2) and in BOCF analyses (Table S3).

**Figure 2:**
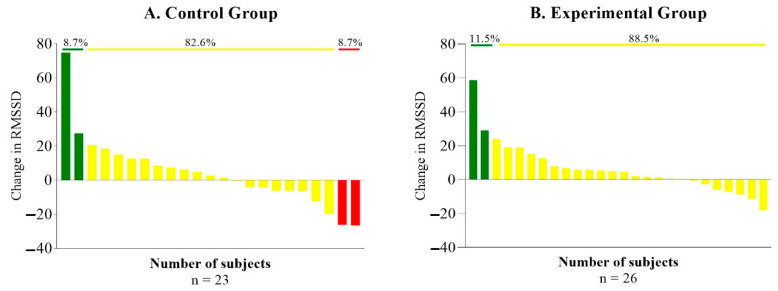
Responders (green line), non-responders (yellow line), and adverse responders (red line) on RMSSD endpoints. RMSSD; root mean square successive difference.

Regarding responders, non-responders, and adverse responders, in the control group we observed significant differences in RMSSD between responders against non-responders and adverse responders (*p* = 0.37 and *p* = 0.002, respectively) and between non-responder and adverse responder (*p* = 0.37). In the exercise group, there was a significant difference in RMSSD between responders and non-responders (*p* = 0.001) Figure 2.

## 4. Discussion

Our cross-sectional analyses revealed that, among the studied HRV-related variables, sample entropy was inversely associated with hsCRP and MPO and that low frequency and high frequency ratio was directly associated with physical fatigue in women with SLE. The secondary analyses of our clinical trial revealed that 12 weeks of progressive aerobic training did not change HRV-derived parameters in comparison to a control group of SLE patients who received recommendations for a healthy lifestyle.

Imbalance in the sympathetic and parasympathetic divisions of the ANS are associated with increased risk of inflammation [8] which could lead to higher cardiovascular risk [41]. In our study, we observed that higher values of hsCRP and MPO were associated with decreased regularity (SampleEn) but not with any other HRV parameter. Elevated hsCRP and MPO levels have been shown to be increased in this population and associated with inflammation [42]. In addition, MPO and hsCRP accurately predicted cardiovascular mortality risk and risk assessment in coronary angiography patients [43]. Several inflammatory pathways seem to be involved in the relationship with HRV. One of the possible explanations could be changes in the activity of the vagal system that modulates the inflammatory response significantly, which can be blocked or enhanced by transmitter substances (i.e., noradrenaline) or by pro-inflammatory cytokines [44]. A decrease in regularity (SampleEn) could be related to the idea proposed by Goldberger et al. [45], in which nonlinear complexity breaks down with aging and disease reducing the individual’s adaptive capabilities. We also found a positive correlation between HRV and IL-6 but not with TNF- α. After adjusting the quantile regression model by age, heart rate, and disease duration we did not find an association between HRV and IL-6. However, it should be noted that both inflammatory markers and ANS have a circadian variation and that the explanatory power of correlating HRV activity and inflammation may be limited by the time frame of the analysis [46]. Given that our HRV data were collected in the afternoon and once at baseline and after the intervention, this could affect our conclusions about these associations.

Regarding PROs, we did not find in our sample associations between HRV and depression, stress or health-related quality of life as previously reported [47]. However, we observed an association between HRV and physical fatigue, as previous findings in other illnesses such as breast cancer [48]. According to Pagani et al. [49], slow autonomic responses to environmental demands or an imbalance between sympathetic and parasympathetic branches may contribute to reduced physical activity, and increased fatigue. It is important to note that fatigue improvements have been described in SLE independently of changes in fitness levels and that fatigue is a multifaceted phenomenon that might be affected by different peripheral and central mechanisms [50]. However, we have observed reductions in general fatigue after our exercise intervention with cardiorespiratory fitness as a mediator [20], which could be related to a better conditioning in these patients.

To the best of our knowledge, no prior research has evaluated the effects of aerobic exercise on HRV in women with SLE. Yorgun et al. [28] studied HRV during 24 h in SLE patients and controls after an exercise stress test finding a higher QT dispersion, along the lines of previous work by Rivera-López et al. [51], and impairments in the autonomic cardiac function in SLE patients compared to controls. A similar study was performed by Bienias et al. [29] controlling the effect of beta-blockers in one of the groups, concluding that impaired heart rate recovery was associated with disease duration and beta-blocker treatment. Our results showed no differences in HRV between groups after an aerobic exercise program. However, as shown in Figure 2, there are some participants that improved their RMSDD after the intervention and, compared to the control group, all participants slightly improved as well even if these differences were not significant. It is important to note that our sample size is small, and we had dropout patients in both groups, although our results were consistent across different sensitivity analyses (Tables S2 and S3). This show that, although our intervention improved CRF in these patients [19], it was not as effective in other secondary parameters such as HRV. Therefore, a more effective or intense intervention program could have had improvements in HRV and other physiological parameters. In fact, HRV as a tool to guide daily training has shown to be superior (at increasing fitness and exercise performance) to other training conventional methods [52].

This study has limitations. First, since our sample size was relatively small, and this study is exploratory and hypotheses-generating in nature, we did not perform corrections for multiple comparisons, which would likely eliminate all the observed associations. Future studies with larger samples should confirm or contrast these findings. Second, only women with mild/inactive disease were included. Therefore, the results are not generalizable to men or even women with medium–high disease. Third, this study comes from the secondary analysis of a non-randomized design, and, despite statistical adjustment, residual confounding cannot be discarded. Four, we did not have a group of healthy subjects performing the exercise program, which would have enabled us to compare the results. However, the study also has some strengths that must be highlighted. First, to our knowledge this is the most comprehensive study done about HRV in women with SLE. Second, we have shown how everyone responded individually to the exercise program based on their HRV.

## 5. Conclusions

Our study suggests that increases in hsCRP and MPO are related to decreased regularity, and that physical fatigue seems to be related to HRV in women with SLE. Additionally, 12 weeks of progressive aerobic training (75 min twice a week) did not produce any changes in HRV derived parameters compared to a usual care control group in women with mild/inactive disease. Future clinical trials with larger sample sizes and a different training program or with higher intensity are needed to enhance our understanding on how HRV could help monitor inflammation in this population; and how they respond to an exercise intervention using HRV as a guideline to prescribe training on a day-to-day basis.

## Figures and Tables

**Table 1:** Baseline characteristics of the study participants.

	All (*n* = 55)	Exercise (*n* = 26)	Control (*n* = 29)	*p*
	Mean (SD)	Mean (SD)	Mean (SD)	
Age, years	43.5 (14.0)	42.9 (15.1)	43.9 (13.3)	0.808
BMI, kg/m^2^	25.4 (4.8)	25.9 (3.4)	25.0 (5.8)	0.491
SBP, mm/Hg	117.5 (10.3)	116.8 (9.9)	118.1 (10.6)	0.653
DBP, mm/Hg	75.3 (9.4)	75.5 (8.7)	75.1 (10.01)	0.843
MBP, mm/Hg	94.6 (8.7)	94.5 (8.3)	94.7 (9.2)	0.937
Mean HR, bpm	76.70 (10.71)	79.11 (9.76)	74.54 (11.23)	0.112
hsCRP, mg/L (median, IQR)	1.6 (2.6–6.5)	2.2 (1.9–7.6)	1.2 (1.5–7.1)	0.218
IL-6, pg/mL (median, IQR)	10.5 (9.4–12.3)	8.2 (7.1–11.7)	11.3 (10.3–14.0)	0.018
TNF-α, pg/mL (median, IQR)	15.6 (15.7–19.8)	16.5 (15.4–21.1)	14.8 (14.3–20.4)	0.385
MPO, ng/mL (median, IQR)	69.6 (79.1–119.6)	60.1 (62.4–126.9)	75.7 (76.3–130.9)	0.385
Smoke (%)	23.6	15.4	31.0	0.237
Menopause (%)	38.2	38.5	37.9	0.968
Dyslipidemia (%)	16.4	19.2	13.8	0.586
Statins (%)	16.4	23.1	10.3	0.203
Immunosuppressants (%)	45.5	46.1	44.8	0.921
Current corticosteroid intake (mg/day)	3.86 (5.1)	4.08 (6.1)	3.70 (4.2)	0.789
Disease duration, years	15.1 (10.1)	14.54 (10.4)	15.6 (9.9)	0.704
Total PA, min/week	94.8 (92.6)	97.5 (95.9)	92.4 (91.1)	0.660
SLEDAI	0.16 (0.764)	0.04 (0.196)	0.28 (1.0)	0.254
SDI	0.42 (1.1)	0.19 (0.63)	0.62 (1.3)	0.145
Psychological Stress (PSS; 0–56; median, IQR)	31.0 (28.9–32.1)	30.0 (27.7–31.6)	31.0 (28.7–33.9)	0.303
Depressive symptoms (BDI-II; 0–63)	12.8 (9.2)	8.0 (6.4–12.7)	17.0 (12.2–19.3)	0.011
Fatigue (MFI-S; 0–20)				
General Fatigue (median, IQR)	15.0 (12.9–15.1)	14.5 (12.1–15.3)	16.0 (12.5–15.9)	0.498
Physical fatigue	12.8 (4.7)	12.4 (4.8)	13.1 (4.7)	0.577
Reduced Activity (median, IQR)	10.0 (8.7–11.5)	8.0 (7.8–11.5)	11.0 (8.4–12.6)	0.741
Reduced Motivation	9.4 (3.7)	8.5 (3.4)	10.1 (3.9)	0.112
Mental Fatigue	12.2 (2.8)	12.04 (3.0)	12.3 (2.6)	0.720
Health-related quality of life (SF-36; 0–00) *				
Physical Component Summary	43.0 (8.2)	45.5 (8.5)	40.6 (7.8)	0.034
Mental Component Summary	44.9 (11.0)	47.5 (11.7)	40.4 (11.0)	0.106

* For SF-36 domains total sample size was *n* = 45 due to missing data. Values are the mean (standard deviation; SD), unless otherwise indicated. BMI, body mass index; DBP, diastolic blood pressure; HR, heart rate; hsCRP, high sensitivity C-reactive protein; IL-6, interleukin-6; mg, milligrams; MBP, mean blood pressure; MPO, myeloperoxidase; PA, physical activity; SBP, systolic blood pressure; SDI, systemic damage index; SLEDAI, systemic lupus erythematosus disease activity index; TNF-α, tumor necrosis factor alpha.

**Table 2:** Baseline heart rate variability (HRV) derived parameters of the study participants.

	All (*n* = 55)	Exercise (*n* = 26)	Control (*n* = 29)	*p*
	Median (IQR)	Median (IQR)	Median (IQR)	
SDNN, ms	19.59 (13.30–25.80)	15.87 (11.34–25.24)	21.42 (14.55–26.36)	0.376
RMSSD, ms	16.20 (11.55–25.07)	14.82 (8.86–24.86)	17.33 (13.61–26.75)	0.292
pNN50 (%)	0.57 (0.21–3.17)	0.42 (0.22–2.78)	0.70 (0.22–3.48)	0.715
LF, ms^2^	164.12 (76.51–340.51)	157.23 (76.51–345.26)	198.18 (76.51–345.26)	0.607
HF, ms^2^	97.20 (39.31–299.42)	93.65 (29.92–334.81)	100.37 (59.40–216.69)	0.607
LFHF	1.57 (0.93–2.81)	1.31 (0.83–3.29)	1.82 (1.08–2.55)	0.980
SD1, ms	11.48 (8.18–17.75)	10.49 (6.27–17.60)	12.27 (9.64–17.60)	0.292
SD2, ms	25.30 (15.54–30.46)	20.86 (18.28–30.42)	25.80 (18.29–30.42)	0.423
SampEn, au	1.70 (1.55–1.83)	1.70 (1.60–1.82)	1.70 (1.51–1.83)	0.692

Values are the median (IQR, interquartile range). HF, high frequency power in absolute value; LF, low frequency power in absolute value; pNN50, percentage of successive normal sinus RR intervals more than 50 ms; RMSSD, root mean square successive difference; SampEn, sample entropy; ms. milliseconds: SD1, standard deviation—poincaré plot crosswise; SD2, standard deviation—poincaré plot lengthwise; SDNN, standard deviation of NN intervals.

**Table 3:** Spearman’s correlations between HRV derived parameters, inflammatory markers, and PROs (*n* = 55).

	hsCRP	IL-6	TNF-α	MPO	SLEDAI	SDI	PSS	BDI	MFI-General Fatigue	MFI-Physical Fatigue	MFI-Reduce Activity	MFI-Reduce Motivation	MFI-Mental Fatigue	SF-36 Physical Component	SF-36 Mental Component
SDNN	−0.05	−0.11	−0.21	0.04	−0.21	−0.14	0.16	−0.11	0.05	−0.14	−0.10	−0.08	−0.06	−0.03	−0.01
RMSSD	−0.09	−0.14	−0.17	−0.01	−0.19	−0.03	0.04	−0.04	0.06	−0.09	0.03	0.03	0.04	0.05	−0.05
pNN50	−0.06	−0.14	−0.14	0.05	−0.09	−0.06	0.16	−0.06	0.10	−0.09	0.05	−0.02	0.04	0.07	−0.04
LF	−0.03	−0.08	−0.23	−0.08	−0.16	−0.17	0.17	−0.13	0.10	−0.08	−0.10	−0.13	−0.05	−0.03	0.01
HF	−0.07	−0.20	−0.23	−0.08	−0.25	−0.15	0.05	−0.14	−0.05	−0.25	−0.13	−0.03	−0.03	0.03	−0.07
LFHF	0.05	0.32 *	0.17	0.20	0.17	0.03	0.08	0.12	0.14	0.30 *	−0.13	−0.05	−0.05	−0.11	0.17
SD1	−0.09	−0.14	−0.17	−0.01	−0.19	−0.03	0.04	−0.04	0.06	−0.09	−0.03	0.04	0.04	0.05	−0.05
SD2	−0.03	−0.09	−0.21	0.09	−0.20	−0.17	0.18	−0.14	0.06	−0.14	0.03	−0.09	−0.09	−0.05	0.00
SampEn	−0.35 **	−0.16	−0.16	−0.32 *	−0.03	−0.05	−0.19	0.15	0.05	0.04	−0.12	0.23	0.23	0.14	0.14

Notes: * *p* < 0.05; ** *p* < 0.01. BDI, Beck depression inventory; HF, high frequency power; hsCRP, high sensitivity C-reactive protein; IL-6, interleukin-6; LF, low frequency power; MFI, multidimension fatigue inventory; MPO, myeloperoxidase; pNN50, percentage of successive normal sinus RR intervals more than 50 ms; PSS, perceived stress scale; RMSSD, root mean square successive difference; SampEn, sample entropy; ms. milliseconds; SD1, standard deviation—poincaré plot crosswise; SD2, standard deviation—poincaré plot lengthwise; SDI, systemic damage index; SDNN, standard deviation of NN intervals; SF-36, short form health survey; SLEDAI, systemic lupus erythematosus disease activity index; TNF-α, tumor necrosis factor alpha.

**Table 4:** Quantile regression analysis evaluating the association between different components of heart rate variability, inflammatory markers, and PROs in women with systemic lupus erythematosus (*n* = 55).

	B	SE	CI 95%		*p*
LFHF					
IL-6	0.48	0.39	−0.31	1.27	0.231
MFI-Physical Fatigue	0.89	0.37	0.15	1.62	0.019
SampEn					
hsCRP	−4.82	1.89	−8.62	−1.03	0.014
MPO	−106.51	37.85	−182.54	−30.50	0.007

hsCRP, high sensitivity C-reactive protein; IL-6, interleukin-6, LFHF, low frequency to high frequency ratio; MFI, multidimensional fatigue inventory; MPO, myeloperoxidase; SampEn, sample entropy; adjusted by age, heart rate and disease duration.

**Table 5:** Per-protocol (primary) analyses assessing the effects of 12-week progressive aerobic exercise on HRV derived parameters in women with systemic lupus erythematosus (participants in the exercise group were included if attendance was ≥75%).

Change from Baseline at Week 12	Exercise (*n* = 21)	Control (*n* = 23)	Median Difference (95% CI)	*p*
	Median (SE)	Median (SE)		
SDNN	2.70 (2.36)	4.18 (2.91)	−1.48 (−12.00 to 6.37)	0.539
RMSSD	2.03 (3.52)	2.75 (4.33)	−0.72 (−12.05 to 9.74)	0.831
pNN50	0.21 (1.93)	0.28 (2.96)	−0.07 (−5.87 to 6.16)	0.960
LF (ms)	2.50 (81.86)	−22.31 (57.00)	24.81 (−142.07 to 169.88)	0.858
HF (ms)	4.76 (98.31)	6.91 (73.40)	−2.15 (−140.79 to 129.24)	0.932
LFHF	−0.12 (1.30)	0.05 (1.01)	−0.17 (−01.45 to 2.30)	0.652
SD1	1.44 (2.49)	1.95 (3.07)	−0.51 (−8.53 to 6.90)	0.831
SD2	3.10 (2.51)	5.22 (3.04)	−2.45 (−11.91 to 6.33)	0.539
SampEn	0.02 (0.07)	0.01 (0.08)	0.01 (−0.31 to 0.23)	0.741

The analyses were adjusted for baseline values, mean heart rate, and age. Values are the median (standard error). HF, high frequency power in absolute value; LF, low frequency power in absolute value; pNN50, percentage of successive normal sinus RR intervals more than 50 ms; RMSSD, root mean square successive difference; SampEn, sample entropy; ms. milliseconds; SD1, standard deviation—poincaré plot crosswise; SD2, standard deviation—poincaré plot lengthwise; SDNN, standard deviation of NN intervals.

## Supplementary Materials

The following are available online at https://www.mdpi.com/1660-4601/17/24/9501/s1, Figure S1: Correlations between HRV derived parameters and inflammatory markers (*n* = 55), Table S1: Spearman’s correlations between HRV derived parameters, inflammatory markers, and PROs (*n* = 55), Table S2: Sensitivity analyses assessing the effects of 12-week progressive aerobic exercise on HRV derived parameters in women with systemic lupus erythematosus (participants in the exercise group were included if attendance ≥90%), Table S3: Sensitivity analyses using baseline-observation carried forward imputation assessing the effects of 12-week progressive aerobic exercise on HRV derived parameters in women with systemic lupus erythematosus.
