# Supplementary material for: Heart Rate Variability in Women with Systemic Lupus Erythematosus: Association with Health-Related Parameters and Effects of Aerobic Exercise
Source: Int J Environ Res Public Health. 2020 Dec 18;17(24):9501. doi: 10.3390/ijerph17249501 (PMC7766283; doi:10.3390/ijerph17249501)
Supplement: Supplementary file 1 [file ijerph-17-09501-s001.pdf]

Table S1. Spearman's correlations between HRV derived parameters, inflammatory markers, and PROs (N= 55).

		**hsCRP**	**IL-6**	**TNF -α**	**MPO**	**SLEDAI**	**SDI**	**PSS**	**BDI**	**MFI-General Fatigue**	**MFI-Physical Fatigue**	**MFI-Reduce Activity**	**MFI-Reduce Motivation**	**MFI-Mental Fatigue**	**SF-36 Physical Component**	**SF-36 Mental Component**
SDNN	r_s	-0.05	-0.11	-0.21	0.04	-0.21	-0.14	0.16	-0.11	0.05	-0.14	-0.10	-0.08	-0.06	-0.03	-0.01
	95% CI	-0.32 to 0.21	-0.37 to 0.16	-0.45 to 0.05	-0.23 to 0.30	-0.45 to 0.06	-0.39 to 0.13	-0.11 to 0.41	-0.37 to 0.16	-0.21 to 0.32	-0.39 to 0.13	-0.36 to 0.17	-0.34 to 0.19	-0.32 to 0.21	-0.31 to 0.27	-0.30 to 0.28
	p	0.69	0.41	0.12	0.76	0.13	0.32	0.24	0.42	0.69	0.32	0.46	0.56	0.68	0.87	0.96
RMSDD	r_s	-0.09	-0.14	-0.17	-0.01	-0.19	-0.03	0.04	-0.04	0.06	-0.09	0.03	0.03	0.04	0.05	-0.05
	95% CI	-0.35 to 0.18	-0.39 to 0.13	-0.42 to 0.10	-0.28 to 0.25	-0.43 to 0.08	-0.29 to 0.24	-0.23 to 0.30	-0.31 to 0.22	-0.20 to 0.32	-0.34 to 0.18	-0.24 to 0.29	-0.24 to 0.29	-0.23 to 0.30	-0.25 to 0.33	-0.34 to 0.24
	p	0.52	0.30	0.21	0.93	0.17	0.82	0.78	0.75	0.64	0.53	0.83	0.83	0.79	0.75	0.73
pNN50	r_s	-0.06	-0.14	-0.17	0.05	-0.09	-0.06	0.16	-0.06	0.10	-0.09	0.05	-0.02	0.04	0.07	-0.04
	95% CI	-0.32 to 0.21	-0.39 to 0.13	-0.42 to 0.10	-0.22 to 0.31	-0.43 to 0.08	-0.32 to 0.21	-0.11 to 0.41	-0.32 to 0.21	-0.17 to 0.36	-0.34 to 0.18	-0.22 to 0.31	-0.28 to 0.25	-0.23 to 0.30	-0.22 to 0.35	-0.32 to 0.26
	p	0.66	0.31	0.21	0.71	0.17	0.66	0.24	0.66	0.47	0.53	0.72	0.90	0.78	0.64	0.81
LF	r_s	-0.03	-0.08	-0.23	0.06	-0.16	-0.17	0.17	-0.13	0.10	-0.08	-0.10	-0.13	-0.05	-0.03	0.01
	95% CI	-0.30 to 0.23	-0.34 to 0.19	-0.46 to 0.04	-0.21 to 0.32	-0.41 to 0.11	-0.42 to 0.10	-0.10 to 0.42	-0.38 to 0.14	-0.17 to 0.36	-0.33 to 0.19	-0.35 to 0.17	-0.38 to 0.14	-0.31 to 0.22	-0.32 to 0.26	-0.28 to 0.30
	p	0.81	0.56	0.10	0.66	0.24	0.21	0.20	0.35	0.45	0.58	0.49	0.35	0.71	0.84	0.95
HF	r_s	-0.07	-0.20	-0.23	-0.08	-0.26	-0.15	0.05	-0.14	-0.05	-0.25	-0.13	-0.13	-0.03	0.03	-0.07
	95% CI	-0.33 to 0.20	-0.44 to 0.07	-0.47 to 0.03	-0.34 to 0.19	-0.52 to 0.03	-0.40 to 0.12	-0.21 to 0.31	-0.39 to 0.13	-0.31 to 0.22	-0.49 to 0.01	-0.38 to 0.14	-0.38 to 0.14	-0.29 to 0.24	-0.27 to 0.31	-0.12 to 0.44
	p	0.60	0.15	0.09	0.58	0.06	0.28	0.70	0.31	0.72	0.06	0.34	0.34	0.86	0.86	0.25
LFHF	r_s	0.05	0.32*	0.17	0.20	0.17	0.03	0.08	0.12	0.14	0.30*	0.10	-0.03	-0.05	-0.11	0.17
	95% CI	-0.21 to 0.31	0.06 to 0.54	-0.10 to 0.42	-0.07 to 0.44	-0.10 to 0.42	-0.24 to 0.29	-0.19 to 0.34	-0.15 to 0.37	-0.13 to 0.39	0.04 to 0.52	-0.17 to 0.35	-0.29 to 0.24	-0.31 to 0.22	-0.39 to 0.18	-0.12 to 0.44
	p	0.70	0.02	0.21	0.15	0.22	0.84	0.55	0.38	0.32	0.03	0.48	0.84	0.70	0.46	0.25
SD1	r_s	-0.09	-0.14	-0.17	-0.01	-0.19	-0.03	0.04	-0.04	0.06	0.03	0.03	0.03	0.04	0.05	-0.05

	95% CI	-0.35 to 0.18	-0.39 to 0.13	-0.42 to 0.10	-0.28 to 0.25	-0.43 to 0.08	-0.29 to 0.24	-0.23 to 0.30	-0.31 to 0.22	-0.20 to 0.32	-0.24 to 0.29	-0.24 to 0.29	-0.24 to 0.29	-0.23 to 0.30	-0.25 to 0.33	-0.34 to 0.24
	p	0.52	0.30	0.21	0.93	0.17	0.82	0.78	0.75	0.64	0.83	0.83	0.83	0.79	0.75	0.73
SD2	r_s	-0.03	-0.09	-0.21	0.09	-0.20	-0.17	0.18	-0.14	0.06	-0.14	-0.15	-0.12	-0.09	-0.05	0.00
	95% CI	-0.29 to 0.24	-0.34 to 0.13	-0.45 to 0.06	-0.18 to 0.25	-0.44 to 0.07	-0.41 to 0.10	-0.09 to 0.42	-0.39 to 0.13	-0.20 to 0.32	-0.39 to 0.13	-0.40 to 0.12	-0.37 to 0.15	-0.34 to 0.18	-0.34 to 0.24	-0.29 to 0.29
	p	0.85	0.53	0.13	0.53	0.14	0.22	0.20	0.32	0.64	0.31	0.29	0.38	0.53	0.73	0.98
SampEn	r_s	-0.35**	-0.16	-0.16	-0.32*	-0.03	0.05	-0.19	0.15	0.05	0.04	0.20	0.20	0.23	0.24	0.14
	95% CI	-0.56 to-0.10	-0.41 to 0.11	-0.18 to 0.34	-0.54 to -0.06	-0.29 to 0.24	-0.22 to 0.31	-0.44 to 0.08	-0.12 to 0.40	-0.22 to 0.31	-0.23 to 0.30	-0.07 to 0.44	-0.07 to 0.44	-0.04 to 0.47	-0.06 to 0.48	-0.15 to 0.42
	p	0.01	0.25	0.53	0.02	0.84	0.71	0.16	0.28	0.73	0.79	0.15	0.14	0.09	0.05	0.35

Notes: *p<0.05; **p<0.01.

r_s, spearman's rho; 95% CI, confidence intervals; BDI, Beck depression inventory; HF, high frequency power; hsCRP, high sensitivity C-reactive protein; IL-6, interleukin-6; LF, low frequency power; MFI, multidimension fatigue inventory; MPO, myeloperoxidase; pNN50, percentage of successive normal sinus RR intervals more than 50 ms; PSS, perceived stress scale; RMSSD, root mean square successive difference; SampEn, sample entropy; ms. milliseconds; SD1, standard deviation – poincaré plot crosswise; SD2, standard deviation – poincaré plot lengthwise; SDI, systemic damage index; SDNN, standard deviation of NN intervals; SF-36, short form health survey; SLEDAI, systemic lupus erythematosus disease activity index; TNF-α, tumor necrosis factor alpha.

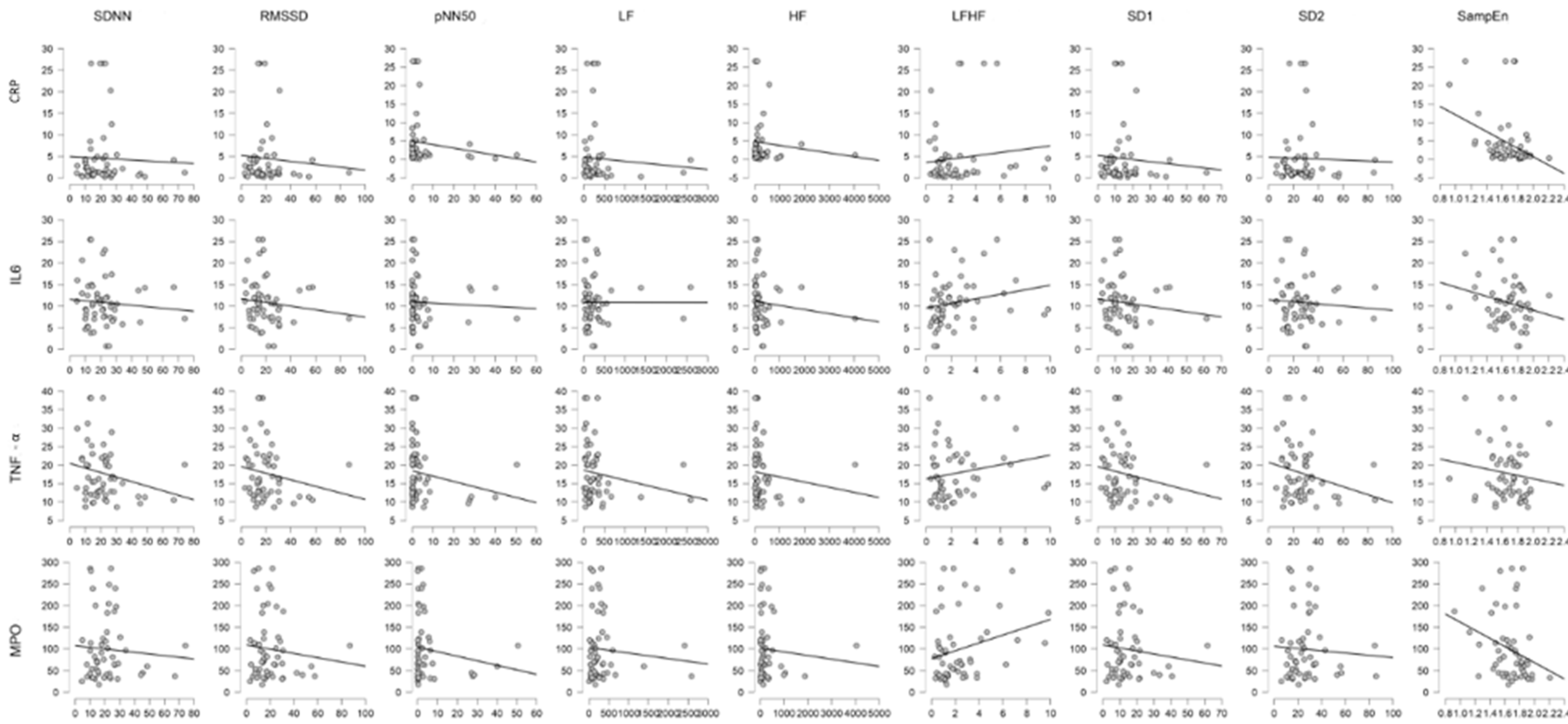

Figure S1. Correlations between HRV derived parameters and inflammatory markers (N=55).

Table S2. Sensitivity analyses assessing the effects of 12-week progressive aerobic exercise on HRV derived parameters in women with systemic lupus erythematosus (participants in the exercise group were included if attendance ≥90%).

Change from baseline at Week 12	Exercise (n = 18)	Control (n = 23)	***Median Difference (95% CI)***	***p***
	Median (SE)	Median (SE)		
SDNN	5.00 (2.64)	4.18 (2.91)	0.82 (-11.08 to 8.20)	0.763
RMSSD	3.67 (4.04)	2.75 (4.33)	0.92 (-14.51 to 12.00)	0.849
pNN50	0.47 (2.19)	0.28 (2.96)	0.19 (-7.93 to 7.78)	0.984
LF (ms)	2.52 (95.49)	-22.31 (57.00)	24.83 (-277.80 to 153.10)	0.561
HF (ms)	10.71 (115.16)	6.91 (73.40)	3.80 (-232.84 to 163.18)	0.723
LFHF	-0.01 (1.47)	0.05 (1.01)	-0.06 (-1.55 to 2.39)	0.670
SD1	2.60 (2.86)	1.95 (3.07)	0.65 (-10.27 to 8.50)	0.850
SD2	4.11 (2.77)	5.22 (3.04)	-1.11 (-12.49 to 6.80)	0.554
SampEn	0.02 (0.08)	0.01 (0.08)	0.01 (-0.25 to 0.37)	0.675

The analyses were adjusted for baseline values, mean heart rate and age. Values are the median (standard error). SDNN, standard deviation of NN intervals; RMSSD, root mean square successive difference; pNN50, percentage of successive normal sinus RR intervals more than 50 ms; LF, low frequency power in absolute value; HF, high frequency power in absolute value; LF/HF, ratio low/high frequency; SD1, standard deviation – poincaré plot crosswise; SD2, standard deviation – poincaré plot lengthwise. SampEn, sample entropy; ms. milliseconds; bpm, beats per minute

Table S3. Sensitivity analyses using baseline-observation carried forward imputation assessing the effects of 12-week progressive aerobic exercise on HRV derived parameters in women with systemic lupus erythematosus.

Change from baseline at Week 12	Exercise (n = 26)	Control (n = 29)	***Mean Difference (95% CI)***	***p***
	Median (SE)	Median (SE)		
SDNN	3.79 (2.00)	0.00 (2.30)	3.79 (-11.59 to 3.27)	0.266
RMSSD	4.70 (2.97)	0.00 (3.44)	4.70 (-10.35 to 8.84)	0.875
pNN50	0.40 (1.75)	0.00 (2.34)	0.40 (-3.39 to 2.00)	0.607
LF (ms)	9.19 (68.53)	0.00 (45.05)	9.19 (-145.40 to 109.40)	0.778
HF (ms)	10.71 (81.25)	0.00 (57.95)	10.71 (-137.69 to 119.44)	0.887
LFHF	0.08 (1.04)	0.00 (0.81)	0.08 (-0.78 to 1.61)	0.491
SD1	3.33 (2.10)	0.00 (2.43)	3.33 (-7.33 to 6.26)	0.875
SD2	3.30 (2.14)	0.00 (2.40)	3.30 (-10.53 to 2.68)	0.238
SampEn	0.02 (0.06)	0.00 (0.06)	0.02 (-0.19 to 0.22)	0.883

The analyses were adjusted for baseline values, mean heart rate and age. Values are the median (standard error). SDNN, standard deviation of NN intervals; RMSSD, root mean square successive difference; pNN50, percentage of successive normal sinus RR intervals more than 50 ms; LF, low frequency power in absolute value; HF, high frequency power in absolute value; LF/HF, ratio low/high frequency; SD1, standard deviation – poincaré plot crosswise; SD2, standard deviation – poincaré plot lengthwise. SampEn, sample entropy; ms. milliseconds; bpm, beats per minute
